# A Local Role for the Small Ribosomal Subunit Primary Binder rpS5 in Final 18S rRNA Processing in Yeast

**DOI:** 10.1371/journal.pone.0010194

**Published:** 2010-04-19

**Authors:** Andreas Neueder, Steffen Jakob, Gisela Pöll, Jan Linnemann, Rainer Deutzmann, Herbert Tschochner, Philipp Milkereit

**Affiliations:** Institut für Biochemie, Genetik und Mikrobiologie, University of Regensburg, Regensburg, Germany; Institute of Protein Research, Russian Academy of Sciences, Russian Federation

## Abstract

*In vivo* depletion of the yeast small ribosomal subunit (SSU) protein S5 (rpS5) leads to nuclear degradation of nascent SSUs and to a perturbed global assembly state of the SSU head domain. Here, we report that rpS5 plays an additional local role at the head/platform interface in efficient SSU maturation. We find that yeast small ribosomal subunits which incorporated an rpS5 variant lacking the seven C-terminal amino acids have a largely assembled head domain and are exported to the cytoplasm. On the other hand, 3′ processing of 18S rRNA precursors is inhibited in these ribosomal particles, although they associate with the putative endonuclease Nob1p and other late acting 40S biogenesis factors. We suggest that the SSU head component rpS5 and platform components as rpS14 are crucial constituents of a highly defined spatial arrangement in the head – platform interface of nascent SSUs, which is required for efficient processing of the therein predicted SSU rRNA 3′ end. Positioning of rpS5 in nascent SSUs, including its relative orientation towards platform components in the head-platform cleft, will depend on the general assembly and folding state of the head domain. Therefore, the suggested model can explain 18S precursor rRNA 3′ processing phenotypes observed in many eukaryotic SSU head assembly mutants.

## Introduction

Ribosomes are large ribonucleoprotein complexes (RNP) which translate mRNA into proteins in all living cells. They consist of two subunits, of which the small ribosomal subunit (SSU) is made of one large ribosomal RNA (rRNA) and more than 20 ribosomal proteins (r-proteins). Atomic resolution crystal structures of prokaryotic ribosomes and cryo-EM analyses of pro- and eukaryotic ribosomes show that large structural domains can be distinguished in the SSU which correspond to the three major secondary structure domains of SSU rRNA: the “head” domain containing the 3′ major domain of SSU rRNA, the “platform” with the SSU rRNA central domain, and the “shoulder” and “foot” containing the SSU rRNA 5′ domain. Shoulder, foot and platform together are also called “body” which is connected by the “neck” with the SSU head domain [1]–[3]. The complete eubacterial SSU, but also the three isolated subdomains can be reconstituted *in vitro* from their purified structural components [4]–[8]. No auxiliary factors are required for SSU *in vitro* assembly, but on the other hand, a hierarchy of individual r-protein rRNA assembly events was observed.

More than 150 non-ribosomal proteins and more than 70 small nucleolar non-coding RNAs (sno-RNAs) engage *in vivo* in maturation of eukaryotic ribosomes [9]. In the model organism *Saccharomyces cerevisiae*, *in vivo* maturation of ribosomes not only involves r-protein - rRNA assembly and rRNA folding, but also nucleo-cytoplasmic transport of nascent ribosomal subunits and an extensive series of precursor rRNA (pre-rRNA) processing and modification events. In addition, pathways exist to efficiently turn over deficient nascent ribosomal subunits [10]–[13]. There are clear indications that several of these aspects of ribosome maturation are strongly interdependent: most of the individual r-protein - rRNA assembly events are required for specific steps in pre-rRNA processing and transport. In addition, inactivation of sno-RNAs which are involved in site specific rRNA modification can lead to perturbations in ribosomal subunit production ([14]–[17] and citations therein). Yeast rpS5, a constituent of the SSU head domain, is one well studied example of a r-protein whose lack of assembly causes distinct SSU maturation phenotypes: *in vivo* depletion of rpS5 leads to nuclear entrapment and degradation of nascent SSUs and to a delay of early nuclear pre-18S rRNA processing events [14]. In addition, similar to what was observed for its prokaryotic counterpart in *in vitro* reconstitution experiments, rpS5 is required *in vivo* for stable assembly of other SSU head proteins. On the other hand, it is nonessential for assembly of many SSU platform and shoulder proteins [4], [18]. The impact of rpS5 on global head domain assembly status, and thereby folding of the 3′ major domain, could well explain several aspects of its various roles in SSU maturation. In the absence of several r-proteins whose stable assembly depends on rpS5, nascent subunits reach the cytoplasm, often with reduced kinetics, but the final cytoplasmic 3′ end processing of SSU pre-rRNA is severely impaired [14], [19]–[21].

Thereby, besides the PIN domain protein Nob1p, which presumably cleaves SSU pre-rRNA at the site defining the mature 3′ end [22]–[24], there is a remarkable number of ribosome biogenesis factors [23], [25], [26] and r-proteins required for this final SSU pre-rRNA processing step. Final SSU pre-rRNA processing correlates in yeast in normal conditions with the ability of nascent SSUs to efficiently engage in translation [27], [28]. The SSU rRNA 3′ end, called site D in yeast, is predicted to be located in mature ribosomes in the neck region close to rpS5 and the platform component rpS14. Point mutations in the C-terminus of rpS14 were shown to delay SSU rRNA 3′ processing [29]. Other r-proteins required for the Nob1p mediated cleavage are localized in mature ribosomes throughout the head domain (rpS3, rpS15, rpS20, rpS29), in the neck (rpS0) and in the head-shoulder interface (rpS2) [14], [19]–[21]. The SSU interaction sites of four other r-proteins (rps10, rpS26, rpS28, rpS31) required for efficient cytoplasmic D site processing are currently unknown [30], [14]. It remains unclear, why so many *in cis* and *in trans* acting factors, which – according to 3D-models – are often predicted to interact with (nascent) SSUs relatively far from site D, are necessary for its efficient processing.

In this work we asked whether rpS5 function in head domain assembly can be uncoupled from potential other, direct roles in the above mentioned aspects of SSU maturation. Based on the existing structural data we created several mutant alleles of RPS5. We screened then for alleles that do not complement the essential functions of rpS5 but partially suppress the rpS5 depletion phenotype. Thereby we indentified a rps5 variant lacking the last seven C-terminal amino acids (subsequently called rpS5-ΔC) which is efficiently incorporated into nascent SSUs and supports establishment of a robust global head domain assembly status. SSUs which incorporated rpS5-ΔC reached the cytoplasm, but showed a strong delay in the final cytoplasmic processing of the SSU rRNA 3′ end. On the other hand, SSUs which incorporated rpS5-deltaC were associated with major non-ribosomal components of late 40S pre-ribosomes. Among them was the presumable endonuclease Nob1p, and other factors which are required for cytoplasmic 3′ processing of pre-rRNA. These data indicate that rpS5 has a dual role in eukaryotic SSU maturation: A first one in global organization of the SSU head domain. And a second, local one, in efficient processing of the SSU rRNA 3′ end. We suggest that the SSU head component rpS5, together with platform components as rpS14, is a crucial constituent of a highly defined spatial arrangement in the head – platform interface of nascent SSUs. We propose that this defined local spatial organisation is required to efficiently process the therein contained SSU rRNA 3′ end. The spatial organisation around rpS5 and rpS14 in the platform – head cleft of nascent SSUs most likely depends on the general assembly and folding status of the head domain. Therefore, the 18S pre-rRNA 3′ processing phenotype observed in many SSU head assembly mutants can be explained by such a model.

## Results

### The seven C-terminal amino acids of rpS5 fulfill an essential function in *S. cerevisiae*

**Figure 1:**
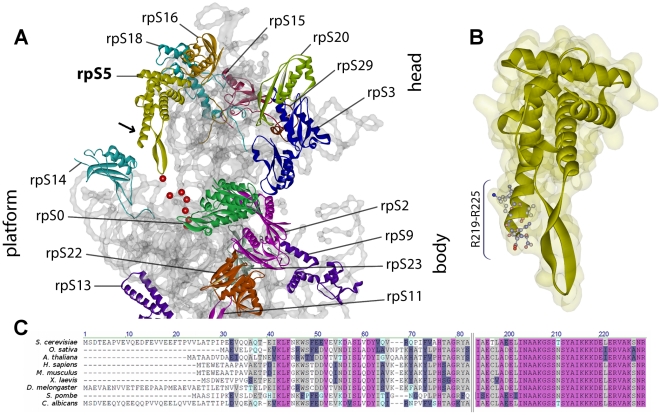
Predicted rpS5 localization and structure and protein sequence conservation. (A) Pseudo-atomic structure model of the eukaryotic small ribosomal subunit (taken from pdb:2ZKQ [3]), cytoplasmic view. Proteins are shown in color, phosphate backbone of 18S rRNA in grey. Positions minus 9 to minus 3 relative to 3′ end of 18S rRNA are highlighted in red. The arrow indicates the C-terminal helix of rpS5. (**B**) Schematic representation of rpS5. The calculated surface is laid underneath. The highly conserved and in rpS5-ΔC variant deleted amino acid residues R219–R225 are shown with side chains. (**C**) Multiple sequence alignment of rpS5 primary structure (AlignX, Vector NTI, Invitrogen, ClustalW algorithm and blosum score-matrix). Sequences are obtained from NCBI database. Colors used are: identical - purple; conserved – grey; block of similar – grey-blue; weakly similar – light-blue.

**Figure 2:**
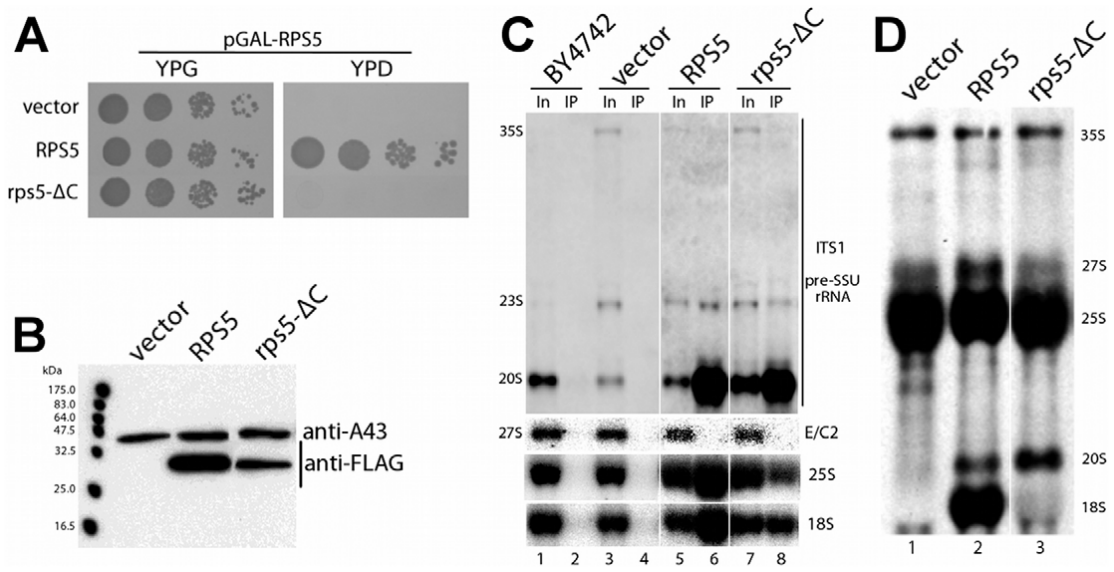
Growth phenotype of rpS5-ΔC and incorporation of rpS5-ΔC into SSU precursors. (**A–D**) All experiments were performed in yeast strain pGAL-RPS5 (ToY323), in which full length rpS5 is encoded under the control of the galactose inducible GAL1 promoter. The strain was either transformed with an empty vector (YEplac195) or vectors ToP996 and ToP1101 coding for FLAG-tagged full length rpS5 or rpS5-ΔC under the control of a constitutive promoter. (**A**) Serial dilutions of the indicated transformants on galactose (YPG) or glucose (YPD) containing plates. Plates were incubated for 3 days at 30°C. (**B–D**) Cells were grown overnight in selective media containing galactose, diluted in YP-galactose (YPG) and subsequently expression of pGAL-RPS5 was shut down for 2 hours in YP-glucose (YPD) medium. (**B**) Western blot analysis of the indicated transformants, using a monoclonal anti-FLAG antibody. A polyclonal Anti-A43 antiserum was used to detect RNA polymerase I subunit A43 as loading control. (**C**) Northern blot analysis of RNA co-immunopurified with the indicated FLAG-tagged rpS5 variants, performed as indicated in Materials and Methods. RNA was extracted from Input (In) and immuno-purified (IP) fractions. Wildtype strain BY4742 served as background control for immuno-purification. Probes used for detection of (pre-) rRNA species are depicted right-hand. (**D**) 5′,6′-[^3^H] uracil metabolic labeling of newly synthesized RNA. Cells were pulsed for 30 minutes at 30°C. Total RNA was extracted and separated by gel electrophoresis, radio-labeled RNA was visualized by fluorography as indicated in Materials and Methods.

*S. cerevisiae* ribosomal protein S5 (rpS5, S7 in prokaryotes) is a structural component of the SSU head domain. Its C-terminal 30 amino-acids are highly conserved in eukaryotes (Fig. 1C). Current (pseudo-) atomic models of pro- and eukaryotic ribosomes predict that the last 18 amino-acids of rpS5 fold in a alpha-helix which does not contact SSU rRNA [1], [3]. The helix rather points away from the SSU head towards rpS14, a component of the SSU platform (Fig. 1A). In an attempt to identify and to subsequently characterize partially functional variants of eukaryotic ribosomal proteins, we cloned the full length RPS5 open reading frame and the RPS5 open reading frame lacking the seven C-terminal amino-acids in fusion with an N-terminal FLAG tag in an *S. cerevisiae* expression vector. When transformed into a yeast strain expressing RPS5 under the control of the galactose inducible GAL1 promoter (strain pGAL-RPS5, [14]), the vector coding for full length rpS5 but neither the vector coding for rpS5 lacking the last seven amino-acids (rpS5-ΔC) nor an empty vector supported yeast growth on glucose containing plates (Fig. 2A). Apparently, although efficiently expressed (Fig. 2B), the mutant allele is not able to fully complement all the essential functions of RPS5.

### rpS5-ΔC is efficiently incorporated into SSU precursors

From SSU 3D-structure model inspection it can be assumed that lack of the seven C-terminal amino-acids should not interfere with rpS5 interaction with SSU rRNA (Fig. 1A/B). In agreement with this it was shown that a C-terminal truncation mutant of E.coli S7 which resembles yeast rpS5 lacking the 11 C-terminal amino acids can still bind *in vitro* to its minimal rRNA binding site [31]. To directly test its in vivo incorporation into yeast ribosomal particles, we affinity purified Flag-tagged rpS5-ΔC and analyzed co-purifying (pre-) rRNA by Northern blotting. RpS5-ΔC showed the same pre-rRNA interaction pattern like wild type rpS5 (Fig. 2C). In addition, co-purification of 18S-rRNA precursors with rpS5-ΔC was comparable to that of full length rpS5, although the variant was apparently less well incorporated into 18S rRNA containing mature ribosomes (compare Fig. 2C lane 6 with lane 8).

### The C-terminal seven amino acids of rpS5 are specifically required for efficient final 3′ end maturation of 18S rRNA precursors

*In vivo* depletion of rpS5 in yeast causes a strong delay, but not complete block of SSU processome dependent nuclear processing at sites A_0_, A_1_, A_2_, resulting in accumulation of 35S- and 23S pre-rRNAs and reduction of steady state levels of 20S pre-rRNA (see processing scheme in Fig. S1, compare in Fig. 2C lane 1 with lane 3, [14]). Accordingly, levels of pulse labeled newly synthesized 20S pre-rRNA, resulting from cleavages at sites A_0_, A_1_ and A_2_, were strongly reduced in strain pGAL-RPS5 shifted to restrictive conditions (Fig. 2D, lane 1). Constitutive co-expression of wild type RPS5 restored synthesis of 18S rRNA (Fig. 2D lane 2). Similarly, expression of the rpS5-ΔC variant led to efficient production of 20S pre-rRNA (Fig. 2D lane 3, compare also steady state levels of 20S pre-rRNA in Fig. 2C, lane 3 and lane 7), apparently abrogating the A_0_, A_1_, A_2_ processing defects seen when wildtype rpS5 is missing. On the other hand, the newly produced 20S pre-rRNA was not efficiently converted into mature 18S rRNA (Fig. 2D, lane 3). Accordingly, the C-terminal 7 amino acids of rpS5 seems to play a crucial role in the endonucleolytic cleavage at site D, converting 20S pre-rRNA into mature 18S rRNA.

### The C-terminal seven amino acids of rpS5 are not strictly required for nuclear export of SSU precursor particles

**Figure 3:**
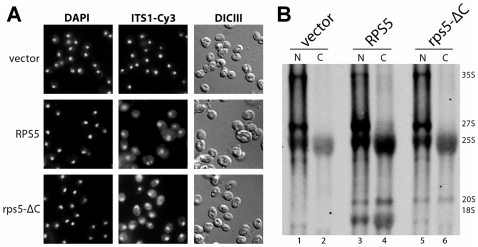
Analyses of nuclear export of SSU precursors containing rpS5-ΔC. All experiments were performed in yeast strain pGAL-RPS5 (ToY323), in which full length rpS5 is encoded under the control of the galactose inducible GAL1 promoter. The strain was either transformed with an empty vector (Yeplac195) or vectors ToP996 and ToP1101 coding for FLAG-tagged full length rpS5 or rpS5-ΔC under the control of a constitutive promoter. (**A**) Steady state distribution of precursor subunits. Cells were grown overnight in selective media containing galactose, diluted in YP-galactose (YPG) and expression of pGAL-RPS5 was shut down for 2 hours in YP-glucose (YPD) medium. Total DNA (DAPI) and rRNA precursors containing ITS1-sequences between site D and A_2_ (ITS1-Cy3, see Fig. S1) were detected as indicated in Materials and Methods. (**B**) Cell fractionation after metabolic RNA labeling. After shut down of pGAL-RPS5 expression, newly synthesized RNA was labeled with [5,6-^3^H] uracil for 20 minutes. Nuclear (N) and cytoplasmic (C) cellular fractions were subsequently separated, RNA was extracted, separated by gel electrophoresis and newly synthesized RNA was visualized by fluorography as described in Materials and Methods.

In wildtype yeast cells, 20S pre-rRNA containing SSU precursors are efficiently exported from the nuclear to the cytoplasmic compartment, where they are converted into 18S rRNA containing mature subunits, which can engage in translation (see processing scheme in Fig. S1). We wondered whether nuclear SSU precursors which incorporated rpS5-ΔC represented a substrate for the nuclear export machinery. Therefore we examined the steady state distribution of SSU precursor particles by FISH (fluorescent *in situ* hybridization) and cell fractionation. In cells depleted for rpS5, a probe against the first internal transcribed spacer sequences (ITS1, see Fig. S1), hybridizing with 20S pre-rRNA and its precursors, detected strictly nucleolar and nuclear signals indicating nuclear retention of SSU precursors ([14] and Fig. 3A, vector). Upon expression of both, rpS5 and its variant rpS5-ΔC, additional cytoplasmic signals were readily detectable (Fig. 3A RPS5 and rps5-ΔC), indicating that SSU precursors reach the cytoplasmic compartment in these strains. Expression of rpS5-ΔC led to an even stronger cytoplasmic ITS1 signal than expression of rpS5 (see also 20S levels in Fig. 2C lanes 5 and 7) underlining a reduction in the conversion to mature 18S rRNA. Cell fractionation after metabolic RNA labeling was used to more directly assess the dynamics of nuclear export of newly synthesized SSU precursors. Newly synthesized RNA was labeled with ^3^H-Uracil and cells were subsequently isolated from a nuclear and a cytoplasmic cellular fraction. In agreement with the FISH experiments no cytoplasmic accumulation of newly synthesized 18S rRNA or its precursors was detectable (Fig. 3B and [14]) when full length rpS5 was depleted. Only minor amounts of nascent nuclear 20S pre-rRNA were evident (compare also with steady state distribution of 20S pre-rRNA in Fig. S2). In contrast, in both strains expressing either rpS5 or its variant rpS5-ΔC, newly synthesized 20S-pre-rRNA reached the cytoplasm (Fig. 3B, for steady state distribution of 20S pre-rRNA see Fig. S2). In agreement with the pulse experiments shown in Fig. 2D, newly synthesized cytoplasmic 20S pre-rRNA was only efficiently converted into 18S rRNA when rpS5, however not rpS5-ΔC was expressed. In addition, while rpS5-ΔC clearly supported nuclear export of SSU precursors, the low ratio of nascent cytoplasmic versus nuclear SSU (pre-) rRNAs argued, that its incorporation in SSU precursors leads either to a delay in their nucleo-cytoplasmic translocation or to a pronounced cytoplasmic destabilization.

### The C-terminal seven amino acids of rpS5 have minor impact on the SSU head domain r-protein assembly status

Similar to what was seen before in *in vitro* reconstitution experiments with prokaryotic SSU components, we previously observed that in *S. cerevisiae* rpS5 is required for stable incorporation of many ribosomal proteins located in the SSU head domain [18]. Establishment of a robust head domain assembly status correlated with efficient cytoplasmic accumulation of SSU precursors. In addition, it was shown that several r-proteins of the head domain are specifically required for cytoplasmic conversion of 20S pre-rRNA into 18S rRNA.

**Figure 4:**
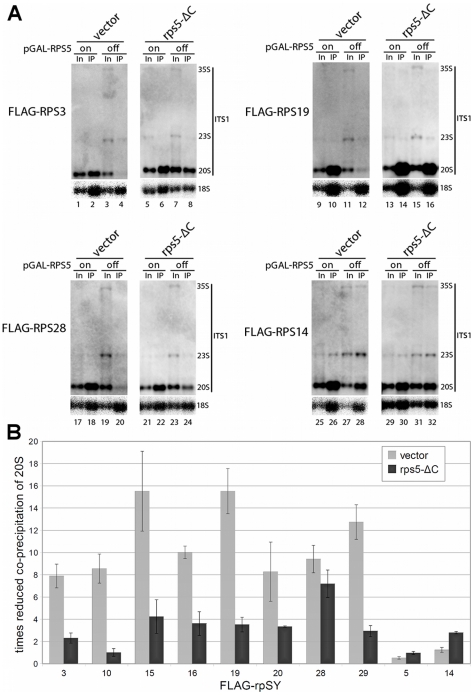
Analyses of r-protein interactions with SSU precursors containing rpS5, rpS5-ΔC or no rpS5. All experiments were performed in yeast strain ToY1659, in which full length rpS5 is encoded under the control of the galactose inducible GAL1 promoter. The strain was transformed with vectors supporting the constitutive expression of the indicated Flag-rpS fusion proteins and, in addition, with an empty vector (YEplac181) or vector ToP1156 coding for HA-tagged rpS5-ΔC under the control of a constitutive promoter. Transformants were grown overnight in selective media containing galactose and on the next day diluted in YP-galactose medium. The cultures were split, one half was further grown in YP-galactose (on), in the other half of the culture expression of pGAL-RPS5 was shut down for 2 hours in YP-glucose medium (off). (**A**) Northern blot analysis of SSU (pre-) RNA co-immunopurifying with the indicated FLAG-tagged rpS in cells expressing rpS5, rpS5-ΔC or no rpS5 (vector) were performed as indicated in Materials and Methods. RNA was extracted from Input (In) and immuno-purified (IP) fractions. Probes used for detection of (pre-) rRNA species are depicted right-hand. See Fig. S3 for the complete set of analyses. (**B**) Quantification of r-protein interaction with SSU precursors containing rpS5, rpS5-ΔC or no rpS5. Each data point was derived from 2 biological replicates. The factor of reduced 20S co-precipitation was calculated as follows: (%IP20S/%IP18S) **on/**(%IP20S/%IP18S) **off**. Quantification was done, using LAS3000, FLA3000 and MultiGauge software (FujiFilm).

Since the rpS5-ΔC variant did not support 18S rRNA production, but could largely relieve the SSU nuclear export phenotype of cells *in vivo* depleted for rpS5, we wondered how rpS5-ΔC affects the assembly status of nascent SSUs. We performed FLAG-epitope affinity purifications of ectopically co-expressed functional FLAG-fusion alleles of various small ribosomal subunit proteins [18] in the absence or presence of rpS5-ΔC. Pre-rRNA co-purification with FLAG-tagged ribosomal proteins was compared before or after shut down of GAL1 promoter driven RPS5 expression for two hours. As observed before (Fig. 2C, lane 3), *in vivo* depletion of rpS5 led to some accumulation of 23S rRNA, and to a reduction of 20S pre-rRNA steady state levels, caused by a delay, but not complete block, of endonucleolytic cleavages at A_0_, A_1_ and A_2_ (Fig. 4A, compare input lanes 1, 9, 17, 25 with input lanes 3, 11, 19, 27, see also Fig. S3). Flag-tagged r-proteins co-purified 20S-pre-rRNA with similar efficiency as mature 18S rRNA when rpS5 was produced (see Fig. 4A, compare input and IP lanes in “on” condition). Upon rpS5 depletion the ratio of 20S pre-rRNA to 18 S rRNA co-purifying with one group of Flag-tagged r-proteins, including rpS10,rpS19, rpS28 and the ones predicted to bind to the SSU head structure (rpS16, rpS3, rpS15, rpS16, rpS20, rpS29), strongly decreased (quantified in Fig. 4B, see also Fig. 4A and Fig. S3). Remarkably, the assembly phenotypes of most of these r-proteins observed upon depletion of rpS5 could be largely relieved by expression of rpS5-ΔC (quantified in Fig. 4B, see also Fig. 4A and Fig. S3). Interestingly, the assembly phenotype of Flag-tagged rpS28 was only slightly suppressed. In conclusion, the last C-terminal seven amino acids of rpS5 seem to have minor impact on global SSU head domain assembly events. They are not strictly required for rpS5 function in nuclear export of SSU precursors, but are crucial for final cytoplasmic 3′ maturation of pre-18S rRNAs.

### Nob1p and other factors required for final 3′ end maturation of pre-18S rRNA are present in SSU precursor particles containing rpS5-ΔC

**Figure 5:**
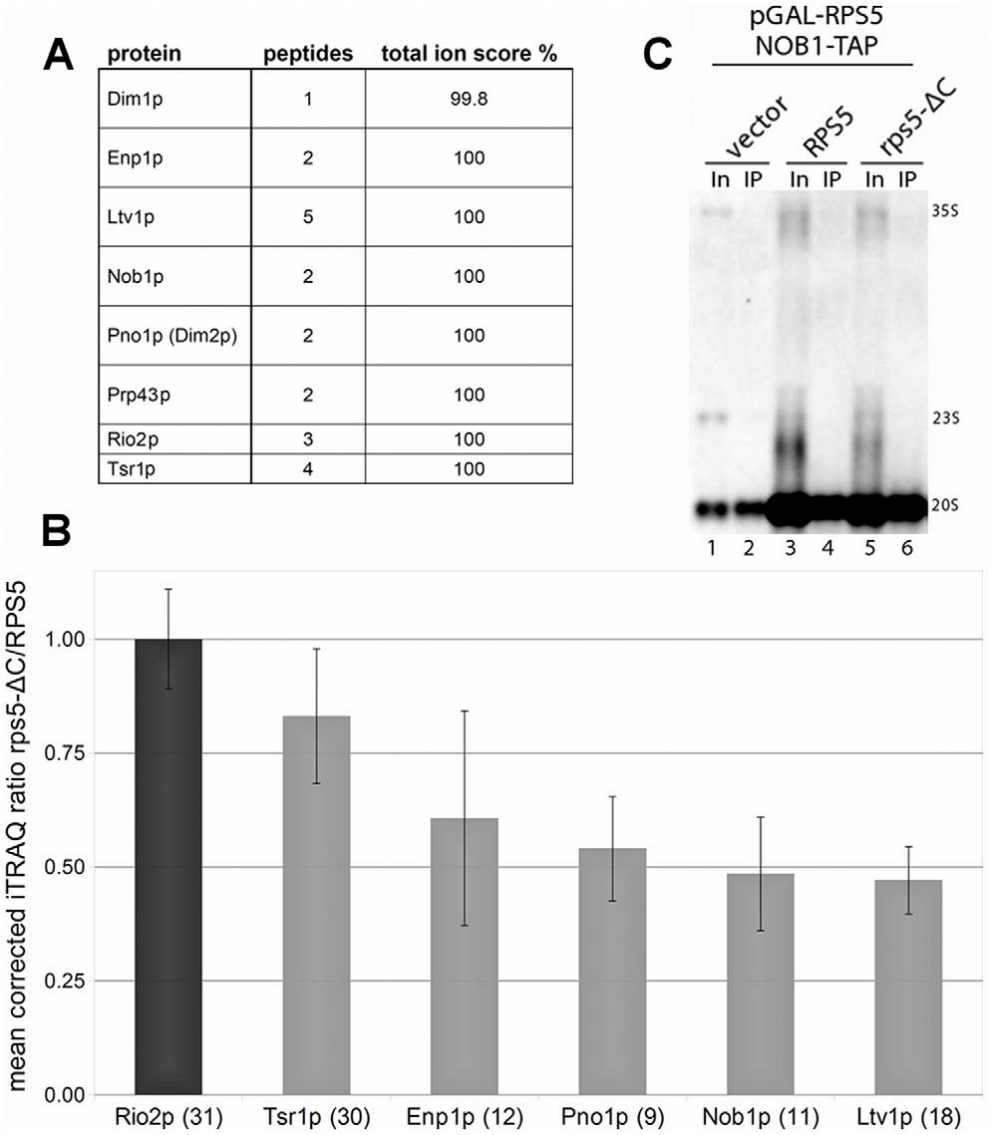
Analyses of the protein composition of SSU precursors containing rpS5-ΔC. (**A–C**) Cells were grown overnight in selective media, diluted in YP-galactose (YPG) and expression of GAL-RPS5 was shut down for 2 hours (3 hours in C) in YP-glucose (YPD) medium. (**A**) FLAG-rpS5-ΔC containing SSU precursors were affinity purified from yeast strain ToY323, transformed with vector ToP1101. Protein content of affinity purified fractions was analyzed by mass spectrometry as indicated in Materials and Methods. The numbers of identified peptides of the indicated proteins together with the total ion scores are indicated. These identified proteins are all major non-ribosomal components of yeast late 40S precursors. In addition to that, one peptide of a non-ribosomal component specific to 60S precursor particles was identified. (**B**) Semi-quantitative comparison of Rio2-TAP associated SSU precursors. Yeast strain ToY1739, in which RPS5 is ectopically expressed under control of a galactose inducible GAL1 promoter and in which Rio2p is expressed as TAP-tag fusion protein, was transformed with vector ToP1162 or vector ToP1156, coding for HA-tagged full length rpS5 or rpS5-ΔC under the control of a constitutive promoter. Rio2-TAP associated SSU precursors were affinity purified from both transformants and their protein composition was compared in a semi-quantitative way by mass spectrometry as described in Materials and Methods. The mean values and standard deviations of three independent biological replicates are shown. The number of analysed tryptic peptides for each protein is given in brackets after the protein name. (**C**) RNA co-immunoprecipitation of TAP-tagged NOB1. Yeast strain ToY1765, in which RPS5 is ectopically expressed under control of a galactose inducible GAL1 promoter and in which Nob1p is expressed as TAP-TAG fusion protein, was transformed with an empty vector (YEplac181), vector ToP1162 or vector ToP1156, coding for HA-tagged full length rpS5 or rpS5-ΔC under the control of a constitutive promoter. Nob1p-TAP was affinity purified and SSU pre-rRNA, contained in input (In) and immuno-purified (IP) fractions was analyzed by Northern blotting as indicated in Materials and Methods.

Recent data suggest that in yeast the formation of the 18S rRNA 3′ end is mediated by the PIN-domain protein Nob1p through an endonucleolytic cleavage event [22]–[24]. In addition to Nob1p and many r-proteins, several ribosome biogenesis factors are specifically required for this last step of pre-18S rRNA processing [25], [26], [23]. Therefore, we wanted to know whether Nob1p or other ribosome biogenesis factors are still incorporated into rpS5-ΔC containing SSU precursor particles. FLAG-rpS5-ΔC and associated RNPs (see Fig. 2C) were purified via an anti-Flag affinity matrix, digested with trypsin, and resulting peptides were separated by nano-HPLC. Mass spectrometric identification of peptides indicated that several biogenesis factors co-purified with FLAG-rpS5-ΔC. In particular Nob1p, the putative endonuclease mediating the 18S rRNA 3′ end processing was identified in the affinity purified fractions (Fig. 5A). To analyze the composition of rpS5-ΔC containing SSU precursors in a more quantitative way, we affinity purified TAP-tagged Rio2p, a constituent of late, 20S pre-rRNA containing SSU precursors ([26], Fig S4), from cells expressing either wild type rpS5 or rpS5-ΔC. Tryptic peptides of these affinity purified fractions were labeled with different iTRAQ reagents, mixed, and analyzed by mass spectrometry. Using this approach we observed relatively minor (less than 50%) differences in the amounts of individual tryptic peptides of Nob1p and of other ribosome biogenesis factors co-purifying with Rio2p-RNPs from cells ectopically expressing either full length rpS5 or rpS5-ΔC after rpS5 depletion (Fig. 5B). Altogether these data indicate that Nob1p, most SSU r-proteins (see above) and the analyzed biogenesis factors can interact with rpS5-ΔC containing SSU precursors. As mentioned before, yeast Nob1p harbors presumably the endonuclease activity converting 20S pre-rRNA into 18S rRNA. Thus we wanted to analyze directly SSU pre-rRNAs co-purifying with TAP-tagged Nob1p in cells expressing either full length rpS5 or rpS5-ΔC. In agreement with the previous data, TAP-tagged Nob1p co-purified similar levels of 20S pre-rRNA, from the respective cellular extracts (Fig. 5C). Accordingly, these results support the assumption that the poor efficiency of pre-18S rRNA maturation in rpS5-ΔC containing SSU precursors is not due to the absence of the endonuclease Nob1p.

## Discussion

Previous work indicated that the absence of rpS5 in yeast strongly affects several aspects of nuclear SSU biogenesis, including stable assembly of other r-proteins, SSU processome mediated pre-rRNA processing events, and pre-SSU stability and nuclear export [18]. Here we provide evidence that most of these phenotypes can be suppressed by expression of a rpS5 variant lacking seven C-terminal amino acids. Nascent SSUs which incorporated rpS5-ΔC accumulate in the cytoplasm and their global assembly status is largely restored. Surprisingly, cytoplasmic 3′ processing of SSU pre-rRNA is strongly delayed in this situation.

Current pseudo-atomic models of eukaryotic ribosomes suggest that the seven C-terminal amino acids of the head domain protein rpS5 are located at the solvent surface of the SSU. They are oriented towards the platform protein rpS14 and thereby constituent of the head-platform cleft in which the SSU rRNA 3′ end is predicted (Fig. 1A). Previous analyses showed that *in vivo* depletion of rpS14 leads to early nuclear blockage in pre-SSU maturation, while mutations in the C-terminus of rpS14 affect cytoplasmic processing of the pre-SSU rRNA 3′ end [29]. Changes in the C-termini of both rpS14 and rpS5 seem not to interfere with pre-SSU binding of Nob1p, the nuclease presumably responsible for this processing step (Fig. 5 and [29]). Thus, these data argue that the two neighboring r-proteins, namely the platform component rpS14 and the head component rpS5 contribute to a local environment in the head-platform cleft around the SSU rRNA 3′ end which is not required for Nob1p binding to its substrate, but to efficiently trigger pre-rRNA cleavage by Nob1p. In agreement with this, Nob1p binds *in vitro* in the absence of other proteinaceous factors with certain specificity pre-rRNA model substrates, while its endonucleolytic activity appears to be rather weak in these conditions [23], [24].

Altogether, these observations lead to some attractive conclusions: Positioning of rpS5 and rpS14 towards each other and towards the SSU rRNA 3′ end will strongly depend on the folding- and assembly- states of the SSU rRNA sub-domains in which they are incorporated. Thereby, their relative position in the platform-head cleft of nascent SSUs mirrors the maturity of these two SSU rRNA sub-domains. In fact, reduced amounts of several SSU head components (rpS15, rpS20, rpS3 [14] and rpS29 (Jakob et al., unpublished observations)) which are located on the other side of the SSU head with respect to rpS5, lead to strong delays in SSU rRNA 3′ processing. We would suggest that lack of stable assembly of these r-proteins, provoked by their direct *in vivo* depletion or by depletion of corresponding assembly factors [32] disturbs general head domain architecture, and thereby has long distance effects on the head-platform interface architecture which negatively influence D-site processing. A case of special interest in this line of evidence are the neck binders rpS0 and rpS2. RpS2 and its prokaryotic homologue S5 bind to the SSU rRNA 5′ domain (shoulder) and, in addition have contact throughout the neck region “thereby stabilizing the conformation of the head with respect to the body” [33] (see also Fig. S5). Strikingly, both absence of rpS2 and rpS0, as well as exchanges of lysines/arginines to alanines in a hairpin of rpS2, expected to participate in interactions stabilizing the global head – body orientation, lead *in vivo* to delays in SSU rRNA 3′ processing (Fig. S5, see also [14]).

Several proteinaceous factors, both SSU structural components and transiently interacting ribosome biogenesis factors, are candidate constituents of the local environment at the head-platform interface important for efficient processing of site D in yeast. Among them are the Nob1p interacting protein Pno1p/Dim2p [34], [35], the rpS14 interacting protein Fap7p [36], and Dim1p, which introduces base modifications near the 18S rRNA 3′ end [37] and whose putative bacterial orthologue KsgA crosslinks nearby with SSU rRNA [38]. More recently, *in vitro* crosslinking experiments suggested sequences around the 18S rRNA 3′ end as one of the major SSU rRNA binding sites of the RNA helicase Prp43p, which in addition shows genetic interactions with Nob1p [39], [23]. Transient binding of all these components might protect the head-platform interface in a chaperone-like way from non-productive interactions with abundant cytosolic (translation related) factors, thereby opening a time window for final folding, and eventually assembly events, which could be further actively promoted by Prp43p′s RNA helicase activity. Remarkably, also in prokaryotes, final *in vivo* maturation of SSUs was suggested to depend on factor mediated assembly and/or folding events in the head-platform interface [40].

In eukaryotes, in addition to rpS5 and rpS14, possible ribosomal constituents of this local environment are rpS10, rpS26, pS28 and rpS31. The location of these in mature SSUs is currently unclear, but they are all required for efficient cytoplasmic processing of 3′ extended SSU rRNA [14], [19]. Interestingly, rpS26 seems to incorporate into nascent subunits at a very late stage [18], and the stable assembly of rpS28 with SSU precursors seems to depend to some extent on the presence of the C-terminus of rpS5 (Fig. 4B). Site directed cross-linking of individual mRNA nucleotides with ribosomal components in translation initiation complexes suggested rpS26 and rpS28 in the rpS5 neighborhood [41].

In addition to its apparent importance in eukaryotic pre-rRNA processing, the platform-head interface, where rpS5 and rpS14 and the SSU rRNA 3′ end are predicted to localize in mature ribosomes, is crucial for ribosome function. The translation initiation factors eIF1A and eIF1 are thought to bind here [42]–[44], rpS5 and rpS14 seem to contribute to the mRNA channel [41]. In addition, the prokaryotic homologue of rpS5, S7, is a functional component of the E-site [45] and the platform is involved in formation of inter-subunit bridges. Accordingly, overexpression of C-terminal mutant alleles of the prokaryotic homologues of rpS5 and rpS14, S7 respectively S11, leads to perturbed ribosome function in *E. coli* [46]. Moreover, truncation of a non-essential, eukaryote specific N-terminal extension of rpS5 affects proper function of yeast ribosomes in translation [47].

It seems therefore an attractive hypothesis that the assembly and folding state of the platform and the head domain, mirrored by the actual conformation of the platform-head interface, is crucial to trigger the removal of 3′ extended spacer rRNA sequences and eventual associated factors. This removal in turn strongly correlates with, or might even improve the capability of nascent SSUs to enter a productive translation cycle. In this way, not yet properly assembled SSUs could be efficiently excluded from the translation process. That could avoid perturbations in both polysomal mRNA translation and further maturation of these SSUs, which might be affected by non-productive interactions with translation factors, mRNA, 60S subunits and tRNA. In support to this, in logarithmically growing yeast cells, the ratio of 20S pre-rRNA versus fully processed 18S rRNA is the highest in 40S fractions while it is strongly reduced in 80S fractions and in polysomal fractions, which contain translational active ribosomes (see amongst others [27], [28]). That means, that 40S subunits, containing fully processed 18S rRNA, engage with clearly higher efficiency in translation than the ones containing 3′ extended 18S pre-rRNA. In several mutants where 3′ SSU rRNA processing is blocked, the pool of matured free 40S subunits is strongly diminished and free 60S subunits, and most likely, free translation initiation factors accumulate. In these situations substantial portions of SSUs containing 3′ extended 18S rRNA seem to associate with initiation factors, 60S subunits and mRNA and are eventual substrates of degradation pathways which detect malfunctioning ribosomes [48]. In conclusion, cytoplasmic 3′ processing of SSU pre-rRNA may not be strictly required for nascent SSUs to engage in translation initiation, but it correlates clearly with full translation competence of newly synthesized SSUs. Therefore we think that not yet fully assembled or folded SSUs are largely excluded from the translation process by the mechanism suggested above due to their kinetic disadvantages in translation initiation when competing with substantial pools of fully matured free SSUs. Defective subunits escaping nuclear degradation pathways [12] might eventually succeed to engage in translation, but will then be eliminated by backup pathways detecting abnormal, stalled ribosomal complexes [13], [48].

## Materials and Methods

### Yeast cell culture, strains and plasmid construction

Standard techniques were used for yeast cell cultivation, cloning of plasmids, transformation and Western blotting [49]–[51]. More detailed information on oligonucleotides, plasmid and strain constructions are indicated in figures S6, S7 and S8 respectively. For detection of Flag-fusion proteins monoclonal antibody M2 (Sigma) was used.

### RNA co-immunoprecipitation experiments

RNA co-immunoprecipitations were carried out as described in [18], with the exception, that no ribonucleoside-vanadyl complex was used in the buffers. Shut down of GAL1-dependent RPSX expression was done for 2 hours by growing the cells in YP glucose, except for ToY1765 where GAL1-dependent RPS5 expression was shut down by 3 hours shift to YP glucose.

### Metabolic RNA labeling

Cells were grown overnight in minimal media containing galactose. The cells were resuspended in YP media containing galactose and grown for 2 to 3 generation times. Expression of GAL1-dependent RPSX expression was shut down by growing the cells in YP glucose media for 2 hours. For each sample 1 OD600 of cells was centrifuged and resuspended in 100 µl buffer R (2% glucose, 1% peptone, 0.6% malt extract, 0.01% yeast extract, 12% mannitol, and 17.8 mM magnesium acetate). 20 µCi of 5′,6′-[^3^H] uracil (GE Healthcare or Perkin Elmer) were added and the cells were incubated at 30°C for 25 to 30 minutes. Total RNA was extracted as described below, and same amounts of radioactivity were loaded onto a denaturing agarose gel (counted with a Packard Tri-Carb 1600TR scintillation counter) transferred onto a membrane (Positive TM, MP-Biomedicals), sprayed with a liquid enhancer (EN3HANCE spray surface, Perkin Elmer) and subjected to fluorography (BioMax MS film, FUJI).

### Cell fractionation with or without metabolic RNA labeling

The procedure was done as described in [15]. In case of pulse labeling experiments same volume percent of nuclear and cytoplasmic fractions were loaded onto a denaturing agarose gel, for the steady state analysis shown in Fig. S2 2.4 times more of nuclear than cytoplasmic fractions were loaded onto a denaturing agorose gel.

### RNA extraction, Northern blotting, and quantification

RNA extractions, northern blotting, and probes were done as described in [14]. Northern blots were analyzed using a FLA3000 (FUJI) or a LAS Reader 3000 (FUJI). Data were quantified using the MultiGauge software (FUJI).

### Fluorescence in situ hybridization

Cells were grown in YP galactose and resuspended in YP glucose media for 2 hours, to shut down expression of GAL1-driven RPS5. Cells were fixed with 4% para-formaldehyde in YP glucose for 30 min at 30°C and were further processed as described in [52]. Images were captured with an Axiovert 200 M Zeiss microscope.

### Purification of (pre-) ribosomal particles

(Pre-) ribosomal particles were either purified by immunoprecipitation of FLAG-epitope or protein-A (TAP-tag) epitope tagged proteins. For FLAG-tagged proteins, the immunoprecipitation was done with anti-FLAG M2 beads (Sigma) for 90 minutes at 4°C. After washing with A200 (200 mM KCl, 20 mM Tris-HCl pH 8.0, 5 mM MgAc, 1 mM DTT, 1 mM PMSF, 2 mM benzamidine, 20 U/ml of RNasin (NEB)) plus Triton (0.2% v/v), the beads were washed twice with 1 ml of desalting buffer AC (0.1 M ammonium acetate pH 7.4; 0.1 mM MgCl2 hexa-hydrate) and the proteins were eluted through a basic pH step in two times 0.5 ml of 0.5 M ammonium hydroxide. The eluted fractions were pooled and lyophilized. For TAP-tagged proteins, immunoprecipitation was carried out as described for FLAG-tagged proteins with following exceptions (see also [53]): washing buffer is buffer MB (A200, supplemented with Triton X-100 (0.5% v/v) and Tween 20 (0.1% v/v)); the precipitation matrix was magnetic beads (1 µm BcMagEpoxy, Bioclone Inc.) coupled to rabbit IgG (Sigma-Aldrich). The beads were pre-washed 3 times with buffer MB. Binding is carried out at 4°C for 1 hour. The samples were washed 3 times with buffer MB and 2 times with buffer AC (see before), each 0.7 ml. Elution is done like described before (see FLAG-tag purification). The eluates were pooled and lyophilized.

### Analysis of protein composition of purified (pre-) ribosomal particles by mass spectrometry

The lyophilized protein purifications were resuspended in 20 µl of 50 mM ammonium carbonate (pH 7.5) and 6 µl trypsin (1 mg/ml, Roche) was added. The proteins were digested overnight at 37°C. Once again the peptides were lyophilized and the pellet was resuspended in 20–50 µl of 0.1% TFA. Tryptic peptides were separated on a nano-flow HPLC system (Dionex) with a Pep-Map C18–reversed phase column (LC-Packings/Dionex). Separation was done with a 5% to 95% gradient of 80% Acetonitril, 0.05% TFA. The fractions were mixed at real time with 5 times volume of MALDI matrix (CHCA α-cyano-4-hydroxycinnamic acid, 2 mg/ml in 70% acetonitril/0.1%TFA) and spotted onto a MALDI sample plate (SS, 192 well, Applied Biosystems) by a Dionex Probot system. The samples were analyzed by a MALDI-TOF/TOF 4800 series (Applied Biosystems). For standardization a Cal 4700 standard peptide mixture was used (Applied Biosystems). Identification of peptides was done by a database search by the mass/ionization rate (MASCOT or NCBI database).

### Comparative analyses of protein content of purified pre-ribosomal particles by mass spectrometry

The lyophilized protein purifications were subjected to tryptic digestion and iTRAQ (Applied Biosystems) labeling according to the manufacturer's instructions and further analyzed as described above for non-labeled tryptic peptides. Identification of peptides was done by a database search (MASCOT or NCBI database) including the relevant peptide modifications (reduction, blocking, labeling). Quantification was done by intensity comparison of the iTRAQ area peaks. The quantitative data were normalized to the ratio of bait protein in the different purifications.

### Structure representation and image preparation

PDB files were obtained from the RCSB protein data bank (www.rcsb.org) and processed using the accelrys® Discovery Studio Visualizer 2.5 software. All images were prepared using the GIMP (GNU Image Manipulation Program) software.

## Supporting Information

Figure S1 Simplified scheme of the small ribosomal subunit rRNA maturation pathway in *S. cerevisiae*.
(0.10 MB DOC)

Figure S2 Steady state analysis of pre-rRNA in subcellular fractions.
(0.16 MB DOC)

Figure S3 Analysis of r-protein interactions with SSU precursors containing rpS5, rpS5-ΔC or no rpS5.
(0.74 MB DOC)

Figure S4 pre-rRNA co-purifying with TAP-tagged Rio2p.
(0.25 MB DOC)

Figure S5 Phenotype analysis of yeast expressing the rpS2-KRRAAA variant.
(3.99 MB DOC)

Figure S6 Oligonucleotides used in this study.
(0.04 MB DOC)

Figure S7 Plasmids used in this study.
(0.07 MB DOC)

Figure S8 Yeast strains used in this study.
(0.03 MB DOC)

## References

[1] Wimberly BT, Brodersen DE, Clemons WM, Morgan-Warren RJ, Carter AP (2000). Structure of the 30S ribosomal subunit.. Nature.

[2] Spahn CMT, Gomez-Lorenzo MG, Grassucci RA, Jørgensen R, Andersen GR (2004). Domain movements of elongation factor eEF2 and the eukaryotic 80S ribosome facilitate tRNA translocation.. EMBO J.

[3] Chandramouli P, Topf M, Ménétret J, Eswar N, Cannone JJ (2008). Structure of the mammalian 80S ribosome at 8.7 A resolution.. Structure.

[4] Sykes MT, Williamson JR (2009). A complex assembly landscape for the 30S ribosomal subunit.. Annu Rev Biophys.

[5] Weitzmann CJ, Cunningham PR, Nurse K, Ofengand J (1993). Chemical evidence for domain assembly of the Escherichia coli 30S ribosome.. FASEB J.

[6] Agalarov SC, Zheleznyakova EN, Selivanova OM, Zheleznaya LA, Matvienko NI (1998). In vitro assembly of a ribonucleoprotein particle corresponding to the platform domain of the 30S ribosomal subunit.. Proc Natl Acad Sci USA.

[7] Samaha RR, O'Brien B, O'Brien TW, Noller HF (1994). Independent in vitro assembly of a ribonucleoprotein particle containing the 3′ domain of 16S rRNA.. Proc Natl Acad Sci USA.

[8] Agalarov SC, Selivanova OM, Zheleznyakova EN, Zheleznaya LA, Matvienko NI (1999). Independent in vitro assembly of all three major morphological parts of the 30S ribosomal subunit of Thermus thermophilus.. Eur J Biochem.

[9] Henras AK, Soudet J, Gérus M, Lebaron S, Caizergues-Ferrer M (2008). The post-transcriptional steps of eukaryotic ribosome biogenesis.. Cell Mol Life Sci.

[10] Fang F, Hoskins J, Butler JS (2004). 5-fluorouracil enhances exosome-dependent accumulation of polyadenylated rRNAs.. Mol Cell Biol.

[11] Kuai L, Fang F, Butler JS, Sherman F (2004). Polyadenylation of rRNA in Saccharomyces cerevisiae.. Proc Natl Acad Sci USA.

[12] Houseley J, Tollervey D (2008). The nuclear RNA surveillance machinery: The link between ncRNAs and genome structure in budding yeast?. Biochimica et Biophysica Acta (BBA) - Gene Regulatory Mechanisms.

[13] Cole SE, LaRiviere FJ, Merrikh CN, Moore MJ (2009). A convergence of rRNA and mRNA quality control pathways revealed by mechanistic analysis of nonfunctional rRNA decay.. Mol Cell.

[14] Ferreira-Cerca S, Pöll G, Gleizes P, Tschochner H, Milkereit P (2005). Roles of eukaryotic ribosomal proteins in maturation and transport of pre-18S rRNA and ribosome function.. Mol Cell.

[15] Pöll G, Braun T, Jakovljevic J, Neueder A, Jakob S (2009). rRNA maturation in yeast cells depleted of large ribosomal subunit proteins.. PLoS ONE.

[16] Liang X, Liu Q, Fournier MJ (2007). rRNA modifications in an intersubunit bridge of the ribosome strongly affect both ribosome biogenesis and activity.. Mol Cell.

[17] Liang X, Liu Q, Fournier MJ (2009). Loss of rRNA modifications in the decoding center of the ribosome impairs translation and strongly delays pre-rRNA processing.. RNA.

[18] Ferreira-Cerca S, Pöll G, Kühn H, Neueder A, Jakob S (2007). Analysis of the in vivo assembly pathway of eukaryotic 40S ribosomal proteins.. Mol Cell.

[19] Tabb-Massey A, Caffrey JM, Logsden P, Taylor S, Trent JO (2003). Ribosomal proteins Rps0 and Rps21 of Saccharomyces cerevisiae have overlapping functions in the maturation of the 3′ end of 18S rRNA.. Nucleic Acids Res.

[20] Léger-Silvestre I, Milkereit P, Ferreira-Cerca S, Saveanu C, Rousselle J (2004). The ribosomal protein Rps15p is required for nuclear exit of the 40S subunit precursors in yeast.. EMBO J.

[21] Ford CL, Randal-Whitis L, Ellis SR (1999). Yeast proteins related to the p40/laminin receptor precursor are required for 20S ribosomal RNA processing and the maturation of 40S ribosomal subunits.. Cancer Res.

[22] Fatica A, Oeffinger M, Dlakic M, Tollervey D (2003). Nob1p Is Required for Cleavage of the 3′ End of 18S rRNA.. Mol Cell Biol.

[23] Pertschy B, Schneider C, Gnadig M, Schafer T, Tollervey D (2009). http://www.jbc.org/content/early/2009/09/29/jbc.M109.040774.abstract.

[24] Lamanna AC, Karbstein K (2009). Nob1 binds the single-stranded cleavage site D at the 3′-end of 18S rRNA with its PIN domain.. Proc Natl Acad Sci USA.

[25] Gelperin D, Horton L, Beckman J, Hensold J, Lemmon SK (2001). Bms1p, a novel GTP-binding protein, and the related Tsr1p are required for distinct steps of 40S ribosome biogenesis in yeast.. RNA.

[26] Vanrobays E, Gelugne J, Gleizes P, Caizergues-Ferrer M (2003). Late cytoplasmic maturation of the small ribosomal subunit requires RIO proteins in Saccharomyces cerevisiae.. Mol Cell Biol.

[27] Udem SA, Warner JR (1973). The cytoplasmic maturation of a ribosomal precursor ribonucleic acid in yeast.. J Biol Chem.

[28] Trapman J, Planta RJ (1976). Maturation of ribosomes in yeast. I Kinetic analysis by labelling of high molecular weight rRNA species.. Biochim Biophys Acta.

[29] Jakovljevic J, de Mayolo PA, Miles TD, Nguyen TM, Léger-Silvestre I (2004). The carboxy-terminal extension of yeast ribosomal protein S14 is necessary for maturation of 43S preribosomes.. Mol Cell.

[30] Lacombe T, García-Gómez JJ, de la Cruz J, Roser D, Hurt E (2009). Linear ubiquitin fusion to Rps31 and its subsequent cleavage are required for the efficient production and functional integrity of 40S ribosomal subunits.. Mol Microbiol.

[31] Robert F, Brakier-Gingras L (2001). Ribosomal protein S7 from Escherichia coli uses the same determinants to bind 16S ribosomal RNA and its messenger RNA.. Nucleic Acids Res.

[32] Schäfer T, Maco B, Petfalski E, Tollervey D, Böttcher B (2006). Hrr25-dependent phosphorylation state regulates organization of the pre-40S subunit.. Nature.

[33] Brodersen DE, Clemons WM, Carter AP, Wimberly BT, Ramakrishnan V (2002). Crystal structure of the 30 S ribosomal subunit from Thermus thermophilus: structure of the proteins and their interactions with 16 S RNA.. J Mol Biol.

[34] Vanrobays E, Gélugne J, Caizergues-Ferrer M, Lafontaine DLJ (2004). Dim2p, a KH-domain protein required for small ribosomal subunit synthesis.. RNA.

[35] Tone Y, Toh-E A (2002). Nob1p is required for biogenesis of the 26S proteasome and degraded upon its maturation in Saccharomyces cerevisiae.. Genes Dev.

[36] Granneman S, Nandineni MR, Baserga SJ (2005). The putative NTPase Fap7 mediates cytoplasmic 20S pre-rRNA processing through a direct interaction with Rps14.. Mol Cell Biol.

[37] Lafontaine D, Delcour J, Glasser AL, Desgrès J, Vandenhaute J (1994). The DIM1 gene responsible for the conserved m6(2)Am6(2)A dimethylation in the 3′-terminal loop of 18 S rRNA is essential in yeast.. J Mol Biol.

[38] Xu Z, O'Farrell HC, Rife JP, Culver GM (2008). A conserved rRNA methyltransferase regulates ribosome biogenesis.. Nat Struct Mol Biol.

[39] Bohnsack MT, Martin R, Granneman S, Ruprecht M, Schleiff E (2009). Prp43 Bound at Different Sites on the Pre-rRNA Performs Distinct Functions in Ribosome Synthesis.. Molecular Cell.

[40] Sharma MR, Barat C, Wilson DN, Booth TM, Kawazoe M (2005). Interaction of Era with the 30S Ribosomal Subunit: Implications for 30S Subunit Assembly.. Molecular Cell.

[41] Pisarev AV, Kolupaeva VG, Yusupov MM, Hellen CUT, Pestova TV (2008). Ribosomal position and contacts of mRNA in eukaryotic translation initiation complexes.. EMBO J.

[42] Lomakin IB, Kolupaeva VG, Marintchev A, Wagner G, Pestova TV (2003). Position of eukaryotic initiation factor eIF1 on the 40S ribosomal subunit determined by directed hydroxyl radical probing.. Genes Dev.

[43] Yu Y, Marintchev A, Kolupaeva VG, Unbehaun A, Veryasova T (2009). Position of eukaryotic translation initiation factor eIF1A on the 40S ribosomal subunit mapped by directed hydroxyl radical probing.. Nucleic Acids Res.

[44] Passmore LA, Schmeing TM, Maag D, Applefield DJ, Acker MG (2007). The eukaryotic translation initiation factors eIF1 and eIF1A induce an open conformation of the 40S ribosome.. Mol Cell.

[45] Devaraj A, Shoji S, Holbrook ED, Fredrick K (2009). A role for the 30S subunit E site in maintenance of the translational reading frame.. RNA.

[46] Robert F, Brakier-Gingras L (2003). A functional interaction between ribosomal proteins S7 and S11 within the bacterial ribosome.. J Biol Chem.

[47] Lumsden T, Bentley AA, Beutler W, Ghosh A, Galkin O (2010). Yeast strains with N-terminally truncated ribosomal protein S5: implications for the evolution, structure and function of the Rps5/Rps7 proteins.. Nucleic Acids Res.

[48] Soudet J, Gélugne J, Belhabich-Baumas K, Caizergues-Ferrer M, Mougin A (2009). http://www.ncbi.nlm.nih.gov/pubmed/19893492.

[49] Sambrook J, Fritsch E, Maniatis T (1989). Molecular Cloning: A Laboratory Manual. 2. ed..

[50] Burke D, Dawson D, Stearns T (2000). Methods in Yeast Genetics, 2000 Edition: A Cold Spring Harbor Laboratory Course Manual. 1. ed..

[51] Longtine MS, McKenzie A, Demarini DJ, Shah NG, Wach A (1998). Additional modules for versatile and economical PCR-based gene deletion and modification in Saccharomyces cerevisiae. Yeast 14: 953-961.. http://dx.doi.org/10.1002/(SICI)1097-0061(199807)14:10<953::AID-YEA293>3.0.CO;2-U.

[52] Léger-Silvestre I, Milkereit P, Ferreira-Cerca S, Saveanu C, Rousselle J (2004). The ribosomal protein Rps15p is required for nuclear exit of the 40S subunit precursors in yeast.. EMBO J.

[53] Oeffinger M, Wei KE, Rogers R, DeGrasse JA, Chait BT (2007). Comprehensive analysis of diverse ribonucleoprotein complexes.. Nat Methods.

