# Supplementary material for: A Local Role for the Small Ribosomal Subunit Primary Binder rpS5 in Final 18S rRNA Processing in Yeast
Source: PLoS One. 2010 Apr 19;5(4):e10194. doi: 10.1371/journal.pone.0010194 (PMC2856670; doi:10.1371/journal.pone.0010194)
Supplement: Figure S1 — Simplified scheme of the small ribosomal subunit rRNA maturation pathway in S. cerevisiae. (0.10 MB DOC) [file pone.0010194.s001.doc]

##
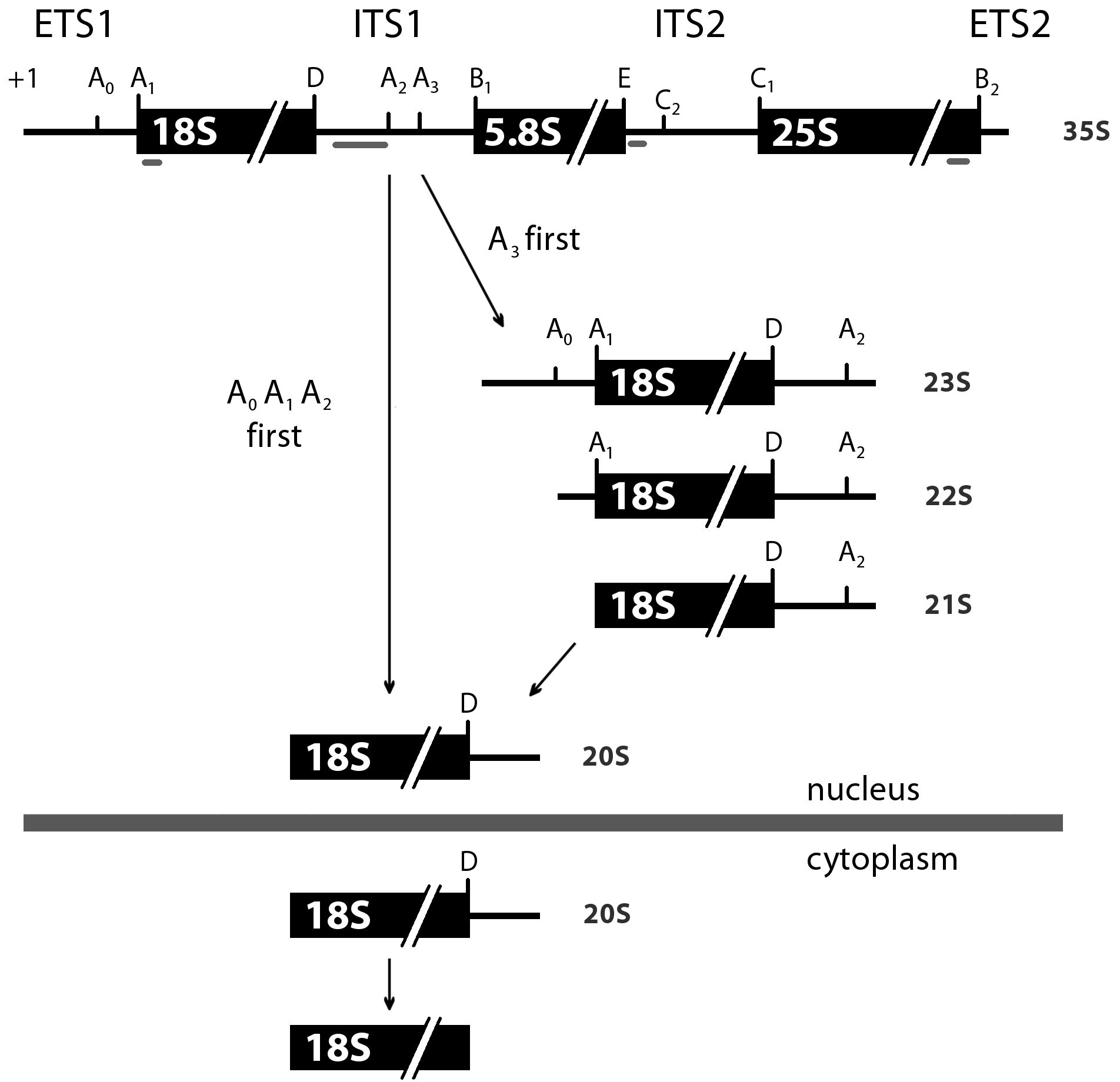


## Figure S1. Simplified scheme of the small ribosomal subunit rRNA maturation pathway in *S. cerevisiae*

The 18S rRNA is transcribed by PolI as a polycistronic precursor transcript (35S), containing in addition the 5.8S and 25S rRNA sequences separated by internal transcribed spacer sequences ITS1 and ITS2. Pre-rRNAs of the large ribosomal subunit are separated from 18S pre-rRNA by cleavage at site A_2_ or A_3_. Precursors are further matured into 20S pre-rRNA, and exported to the cytoplasm. Final processing of 20S to 18S rRNA through endonucleolytic cleavage at site D takes place in the cytoplasm. Regions where probes used in this study hybridize with are indicated by black bars.
