# Supplementary material for: A Local Role for the Small Ribosomal Subunit Primary Binder rpS5 in Final 18S rRNA Processing in Yeast
Source: PLoS One. 2010 Apr 19;5(4):e10194. doi: 10.1371/journal.pone.0010194 (PMC2856670; doi:10.1371/journal.pone.0010194)
Supplement: Figure S2 — Steady state analysis of pre-rRNA in subcellular fractions. (0.16 MB DOC) [file pone.0010194.s002.doc]

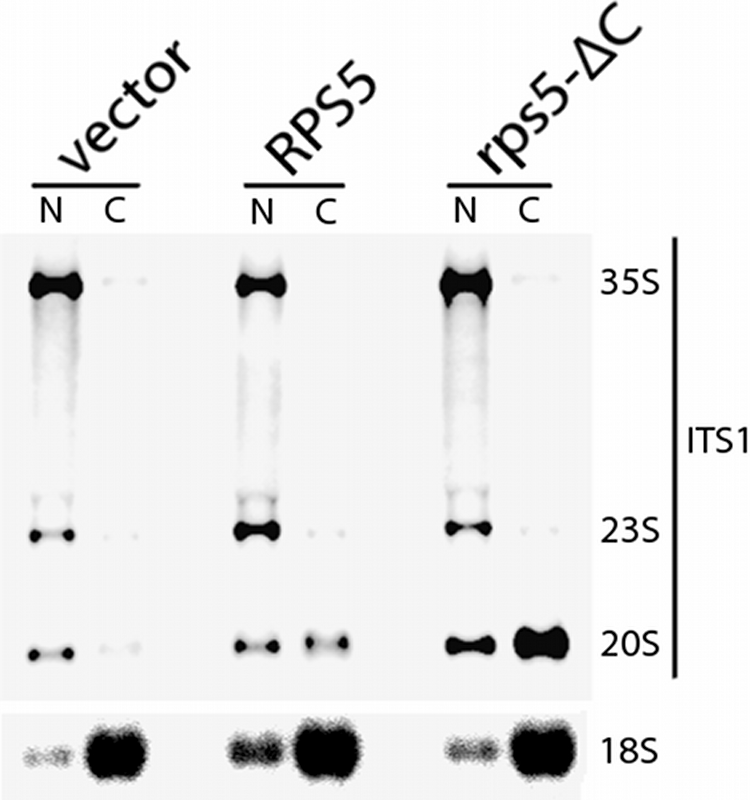


## Figure S2. Steady state analysis of pre-rRNA in subcellular fractions

Yeast strain ToY323, in which full length rpS5 is encoded under control of the galactose inducible GAL1 promoter was transformed with the empty vector YEplac195, vector ToP996 or ToP1101, coding for FLAG-tagged RPS5 or rpS5‑ΔC under the control of a constitutive promoter. Cells were grown overnight in selective media containing galactose, diluted in YP-galactose (YPG) and expression of pGAL-RPS5 was shut down for 1.5 hours in YP-glucose medium (YPD). Cells were spheroblasted and subsequently fractionated in nuclei and cytoplasm. RNA was extracted and 2.4 times more volume of nuclear than cytoplasmic fractions were separated by gel electrophoresis and analyzed by northern blotting. Probes for detection of rRNA species are depicted right-hand. N – nuclear fraction; C – cytoplasmic fraction.
