# Supplementary material for: A Local Role for the Small Ribosomal Subunit Primary Binder rpS5 in Final 18S rRNA Processing in Yeast
Source: PLoS One. 2010 Apr 19;5(4):e10194. doi: 10.1371/journal.pone.0010194 (PMC2856670; doi:10.1371/journal.pone.0010194)
Supplement: Figure S3 — Analysis of r-protein interactions with SSU precursors containing rpS5, rpS5-ΔC or no rpS5. (0.74 MB DOC) [file pone.0010194.s003.doc]

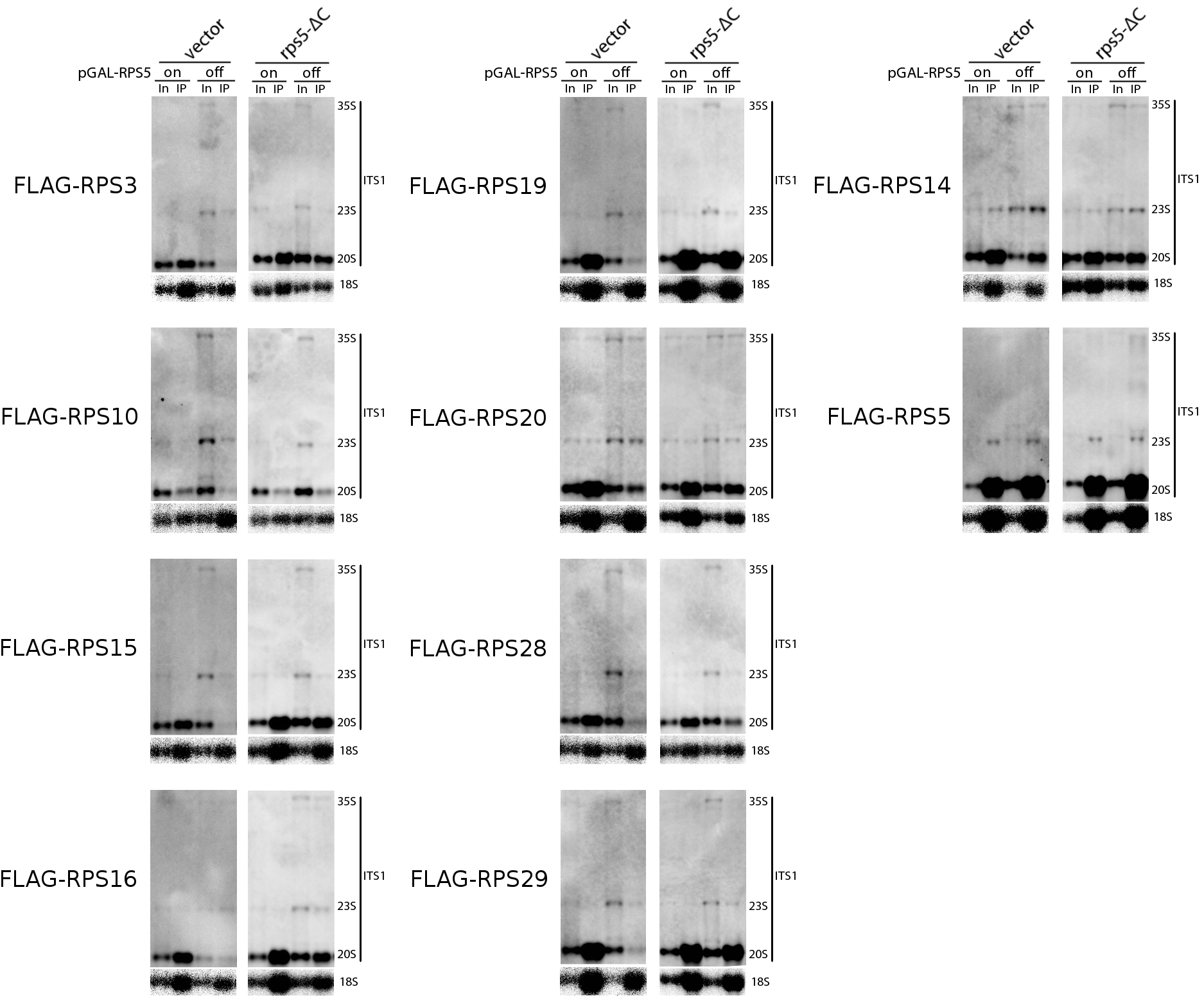


## Figure S3. Analysis of r-protein interactions with SSU precursors containing rpS5, rpS5‑ΔC or no rpS5

All experiments were performed in yeast strain ToY1659, in which full length rpS5 is encoded under the control of the galactose inducible GAL1 promoter. The strain was transformed with vectors supporting the constitutive expression of the indicated Flag‑rpS fusion proteins and, in addition, with an empty vector (YEplac181) or vector ToP1156 coding for HA-tagged rpS5‑ΔC under the control of a constitutive promoter. Transformants were grown overnight in selective media containing galactose and on the next day diluted in YP-galactose medium. The cultures were split, one half was further grown in YP-galactose (on), in the other half of the culture expression of pGAL-RPS5 was shut down for 2 hours in YP-glucose medium (off).

Northern blot analysis of SSU (pre-)RNA co-immunopurifying with the indicated FLAG-tagged rpS in cells expressing rpS5, rpS5-ΔC or no rpS5 were performed as indicated in experimental procedures. RNA was extracted from Input (In) and immuno-purified (IP) fractions. Probes used for detection of (pre-) rRNA species are depicted right-hand.
