# Supplementary material for: A Local Role for the Small Ribosomal Subunit Primary Binder rpS5 in Final 18S rRNA Processing in Yeast
Source: PLoS One. 2010 Apr 19;5(4):e10194. doi: 10.1371/journal.pone.0010194 (PMC2856670; doi:10.1371/journal.pone.0010194)
Supplement: Figure S4 — pre-rRNA co-purifying with TAP-tagged Rio2p. (0.25 MB DOC) [file pone.0010194.s004.doc]

##
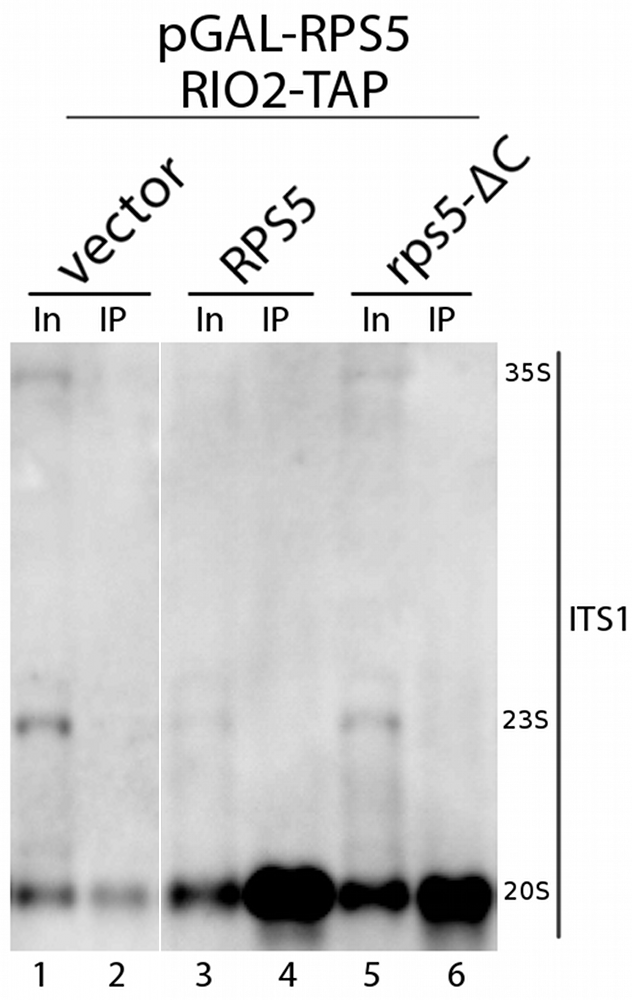
Figure S4. pre-rRNA co-purifying with TAP-tagged Rio2p

Yeast strain ToY1739, in which RPS5 is ectopically expressed under control of a galactose inducible GAL1 promoter and in which Rio2p is expressed as TAP‑tag fusion protein, was transformed with an empty vector YEplac181, vector ToP1162, or vector ToP1156, coding for HA-tagged full length rpS5 or rpS5‑ΔC under the control of a constitutive promoter. Transformants were grown overnight in selective media containing galactose, diluted in YP-galactose (YPG) and expression pGAL-RPS5 was shut down for 2 hours in YP-glucose medium (YPD). Rio2p-TAP was affinity purified and SSU pre-rRNA contained in Input (In) and immuno-purified (IP) fractions was analyzed by Northern blotting as indicated in experimental procedures. Reduced incorporation of Rio2p in SSU-precursors lacking rpS5 will be described in more detail elsewhere.
