# Supplementary material for: A Local Role for the Small Ribosomal Subunit Primary Binder rpS5 in Final 18S rRNA Processing in Yeast
Source: PLoS One. 2010 Apr 19;5(4):e10194. doi: 10.1371/journal.pone.0010194 (PMC2856670; doi:10.1371/journal.pone.0010194)
Supplement: Figure S5 — Phenotype analysis of yeast expressing the rpS2-KRRAAA variant. (3.99 MB DOC) [file pone.0010194.s005.doc]

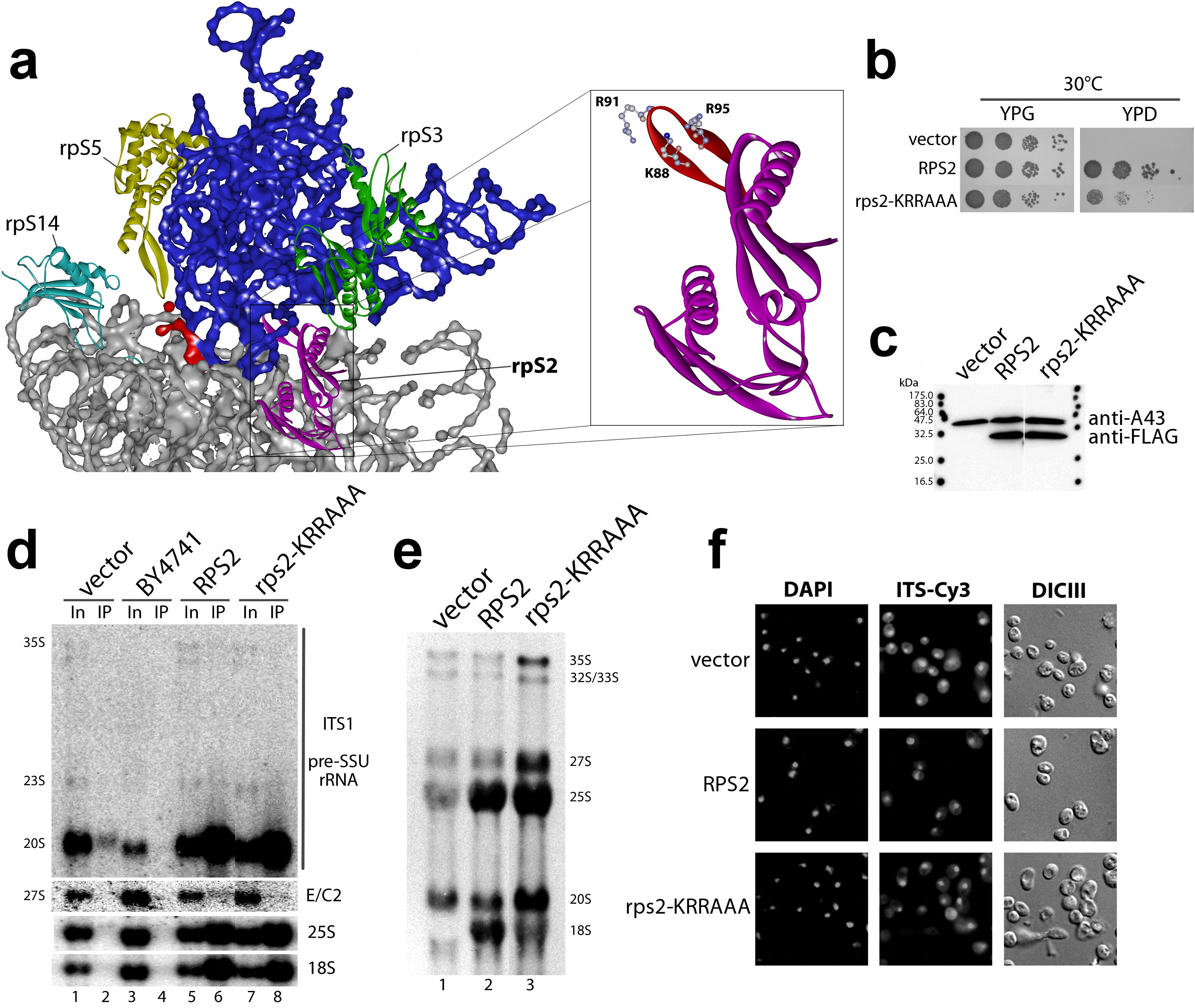


## Figure S5. Phenotype analysis of yeast expressing the rpS2-KRRAAA variant

(**A**) Predicted localization of rpS2 in the small ribosomal subunit (taken from pdb:2ZKQ [3]). Proteins are shown in color, the 3’-major head domain is shown in blue, the modeled nucleotides after helix 45 are shown in red and other 18S rRNA is grey. The hairpin of rpS2 which was mutated in the following experiments is highlighted.

(**B-F**) All experiments were performed in yeast strain pGAL-RPS2 (ToY286), in which full length rpS2 is encoded under the control of the galactose inducible GAL1 promoter. The strain was either transformed with an empty vector (YEplac195) or vectors ToP993 and ToP1107, coding for FLAG-tagged full length rpS2 or a rpS2 variant in which lysine 88, arginine 91 and arginine 95 are replaced by alanines, hereafter called rpS2-KRRAAA.

(**B**) Serial dilutions of the indicated transformants on galactose (YPG) or glucose (YPD) containing plates. Plates were incubated for 3 days at 30°C.

(**C-E**) Cells were grown overnight in selective media containing galactose, diluted in YP-galactose (YPG) and subsequently expression of pGAL-RPS2 was shut down for 2 hours in YP-glucose (YPD) medium.

(**C**) Western blot analysis of the indicated transformants, using a monoclonal anti-FLAG antibody. A polyclonal Anti-A43 antiserum was used to detect RNA polymerase I subunit A43 as loading control.

(**D**) Northern blot analysis of RNA co-immunopurifying with the indicated FLAG‑tagged rpS2 variants, performed as indicated in experimental procedures. RNA was extracted from Input (In) and immuno-purified (IP) fractions. Wildtype strain BY4741 served as background control for immuno-purification. Probes used for detection of (pre-) rRNA species are depicted right-hand.

(**E**) 5’,6’-[^3^H] uracil metabolic labeling of neo-synthesized RNA. Cells were pulsed for 30 minutes at 30°C. Total RNA was extracted and separated by gel electrophoresis, radio-labeled RNA was visualized by fluorography as indicated in experimental procedures.

(**F**) Steady state distribution of precursor subunits. Total DNA (DAPI) and rRNA pre-cursors containing ITS1-sequences between site D and A_2_ (ITS1-Cy3, see Fig. S1) were detected as indicated in experimental procedures.
