# Supplementary material for: A Local Role for the Small Ribosomal Subunit Primary Binder rpS5 in Final 18S rRNA Processing in Yeast
Source: PLoS One. 2010 Apr 19;5(4):e10194. doi: 10.1371/journal.pone.0010194 (PMC2856670; doi:10.1371/journal.pone.0010194)
Supplement: Figure S6 — Oligonucleotides used in this study. (0.04 MB DOC) [file pone.0010194.s006.doc]

Table S6: oligonucleotides used in this study

| **ToO** | **name** | **sequence (5’🡪3’)** |
| --- | --- | --- |
| 205 | 18S | CATGGCTTAATCTTTGAGAC |
| 210 | E/C2 | GGCCAGCAATTTCAAGTTA |
| 212 | 25S | CTCCGCTTATTGATATGC |
| 378 | ITS1-forward | GTTTTGGCAAGAGCATGAGAGC |
| 441 | T7-prom-ITS1-revers | TAATACGACTCACTATAGGGTGTATTGAAACGGTTTTAATTGTCC |
| 466 | RPS5 GAL F | CGCCGCGGATCCATGTCTGACACCGAAGCT |
| 488 | GalRPS2_Rev_PstI | GCAAAACTGCAGTAAAATTTTGATCTATTG |
| 491 | GalRPS2_F_Bam | CGCCGCGGATCCATGTCTGCTCCAGAAGCT |
| 906 | 3xHA-KpnI-Up | GATCTATGTACCCATACGATGTTCCTGACTATGCGTATCCCTATGACGTCCCGGACTATGCATATCCTTATGACGTTCCAGATTACGCTGGTACCG |
| 907 | 3xHA-KpnI-Do | GATCCGGTACCAGCGTAATCTGGAACGTCATAAGGATATGCATAGTCCGGGACGTCATAGGGATACGCATAGTCAGGAACATCGTATGGGTACATA |
| 943 | +Frame-2xHA-Up | GATCTTTACCCATACGATGTTCCTGACTATGCGTATCCCTATGACGTCCCGGACTATGCAGTCGACG |
| 944 | +Frame-2xHA-Do | GATCCGTCGACTGCATAGTCCGGGACGTCATAGGGATACGCATAGTCAGGAACATCGTATGGGTAAA |
| 947 | Yc-KpnI-Up | GATCTGGTACCGGATCCTCTAGAGTCGACCTGCA |
| 948 | Yc-KpnI-Do | GGTCGACTCTAGAGGATCCGGTACCA |
| 1880 | nat1_for | CGCGCTAGCTTAATTAAGGCGCGCCAGATC |
| 1881 | nat1_rev | GCGGCTAGCATTACAACAGGTGTTGTCCTC |
| 1913 | RPS2-KRRAAA_for | CAGTTCAAGCGCAAACCGCAGCCGGTC |
| 1914 | RPSS2-KRRAAA_rev | TGCGGTTTGCGCTTGAACTGGCTTGATG |
| 1939 | RPS5delC_rev | GCGCTGCAGTTATTCCAATTCATCCTTCTTC |
| 2221 | NOB1_TAP_f | GAAGCAGCATAACGTCCGCATTGGTAAGGGAAGGTACGTCAACAGTTCCAAAAGGAGAAGTTCCATGGAAAAGAGAAG |
| 2222 | NOB1_TAP_r | GAAAAAGAAAAAGGGCAGCTGCCAACTAGTACACACTACACAGATATTTATGAAAAACATACGACTCACTATAGGG |
| 2316 | RIO2-pBS1479-INT-UP | GGTGTTGAAAATCTAAAAATGGATAAACTAGGAAACTATATACTAGAGTCCATGGAAAAGAGAAG |
| 2317 | RIO2-pBS1479-INT-DO | GGATAACAACTTGATTATTTGCGGCCATTTATGCAGTCGTCTAAACTAAATACGACTCACTATAGGG |
