# Supplementary material for: A Local Role for the Small Ribosomal Subunit Primary Binder rpS5 in Final 18S rRNA Processing in Yeast
Source: PLoS One. 2010 Apr 19;5(4):e10194. doi: 10.1371/journal.pone.0010194 (PMC2856670; doi:10.1371/journal.pone.0010194)
Supplement: Figure S7 — Plasmids used in this study. (0.07 MB DOC) [file pone.0010194.s007.doc]

Table S7: plasmids used in this study

| **ToP** | **name** | **marker** | **features** | **origin** | **cloning strategy** |
| --- | --- | --- | --- | --- | --- |
| 48 | YCplac22-pGAL | AmpR, TRP1 | CEN4, ARS1 | this study | BamHI/EcoRI fragment containing GAL1/GAL10 promoter was cloned into YCplac22 (Gietz and Sugino, 1988) |
| 90 | YEplac181 | AmpR, LEU2 | 2µ | Gietz and Sugino, 1988 |  |
| 97 | pBS1539 | AmpR |  | CellZome |  |
| 255 | YCplac111-pGAL-RPS2 | AmpR, LEU2 | CEN4, ARS1 | Ferreira-Cerca et.al., 2005 |  |
| 259 | YCplac111-pGAL-RPS5 | AmpR, LEU2 | CEN4, ARS1 | Ferreira-Cerca et.al., 2005 |  |
| 322 | YEplac195 | AmpR, URA3 | 2µ | Gietz and Sugino, 1988 |  |
| 349 | YEplac195-pRPS28-FLAG | AmpR, URA3 | 2µ | Ferreira-Cerca et.al., 2007 |  |
| 430 | pRPS28-FLAG-RPS16^*^ | AmpR, URA3 | 2µ | Ferreira-Cerca et.al., 2007 |  |
| 487 | pRPS28-RPS24-FLAG^*^ | AmpR, URA3 | 2µ | Ferreira-Cerca et.al., 2007 |  |
| 584 | Yep-pRPS28KpnI-Ct-FLAG | AmpR, URA3 | 2µ | this study | ToO947 and ToO948 were hybridized and inserted with BamHI/PstI into ToP487. Resulting clones were sequenced to confirm correct identity . |
| 602 | pRPS28-FLAG-5xHA-RPS16 | AmpR, URA3 | 2µ | this study | ToO906 and ToO907 were hybridized and cloned with BamHI into ToP430. Then, ToO943 and ToO944 were hybridized and cloned with BamHI in the resulting plasmid. Resulting clones were sequenced to confirm correct identity . |
| 616 | YCplac111GAL-RPS27-SE | AmpR, LEU2 | CEN4, ARS1 | Ferreira-Cerca et.al., 2005 |  |
| 621 | Yep-pRPS28-5xHA-RPS16 | AmpR, URA3 | 2µ | this study | KpnI/PstI fragment of ToP602 was cloned into ToP584 |
| 638 | pRPS28-Nt-5xHA-RPS27-SE | AmpR, URA3 | 2µ | this study | BamHI/PstI fragment from ToP616 was cloned into ToP621 |
| 771 | YEplac181-pRPS28-5xHA | AmpR, LEU2 | 2µ | this study | EcoRI/BamHI fragment from ToP638 was cloned into ToP90 |
| 993 | YEplac195-pRPS28-FLAG-RPS2^*^ | AmpR, URA3 | 2µ | Ferreira-Cerca et.al., 2007 |  |
| 994 | YEplac195-pRPS28-FLAG-RPS3^*^ | AmpR, URA3 | 2µ | Ferreira-Cerca et.al., 2007 |  |
| 996 | YEplac195-pRPS28-FLAG-RPS5^*^ | AmpR, URA3 | 2µ | Ferreira-Cerca et.al., 2007 |  |
| 1000 | YEplac195-pRPS28-FLAG-RPS10A^*^ | AmpR, URA3 | 2µ | Ferreira-Cerca et.al., 2007 |  |
| 1003 | YEplac195-pRPS28-FLAG-RPS14A^*^ | AmpR, URA3 | 2µ | Ferreira-Cerca et.al., 2007 |  |
| 1004 | YEplac195-pRPS28-FLAG-RPS15^*^ | AmpR, URA3 | 2µ | Ferreira-Cerca et.al., 2007 |  |
| 1005 | YEplac195-pRPS28-FLAG-RPS16^*^ | AmpR, URA3 | 2µ | Ferreira-Cerca et.al., 2007 |  |
| 1008 | YEplac195-pRPS28-FLAG-RPS19^*^ | AmpR, URA3 | 2µ | Ferreira-Cerca et.al., 2007 |  |
| 1009 | YEplac195-pRPS28-FLAG-RPS20^*^ | AmpR, URA3 | 2µ | Ferreira-Cerca et.al., 2007 |  |
| 1015 | YEplac195-pRPS28-FLAG-RPS28^*^ | AmpR, URA3 | 2µ | Ferreira-Cerca et.al., 2007 |  |
| 1016 | YEplac195-pRPS28-FLAG-RPS29^*^ | AmpR, URA3 | 2µ | Ferreira-Cerca et.al., 2007 |  |
| 1060 | pYM17 | AmpR |  | Janke et al., 2004 |  |
| 1101 | YEplac195-pRPS28-FLAG-rps5-ΔC | AmpR, URA3 | 2µ | this study | PCR product from genomic DNA with ToO466 and ToO1939 was cloned with BamHI/PstI into ToP349 |
| 1107 | YEplac195-pRPS28-FLAG-rps2-KRRAAA | AmpR, URA3 | 2µ | this study | Mutations are introduced by ToO1913 and ToO1914. The PCR products from genomic DNA of ToO491 / ToO1914 and ToO488 / ToO1913 were used as templates for a second PCR with ToO488 and ToO491. The resulting fragment was cloned BamHI/PstI into ToP349. Resulting clones were sequenced to confirm correct identity . |
| 1155 | YCplac22-pGAL-RPS5 NAT1 | AmpR, TRP1 | CEN4, ARS1 | this study | Open reading frame of ToP259 was subcloned into ToP48 and the PCR product of ToO1880 and ToO1881 from pYM17 (ToP1060) was inserted with NheI |
| 1156 | YEplac181-pRPS28-5xHA-rps5-ΔC** | AmpR, LEU2 | 2µ | this study | Open reading frame of ToP1101 was subcloned with BamHI/PstI into ToP771 |
| 1162 | YEplac181-pRPS28-5xHA-RPS5* | AmpR, LEU2 | 2µ | this study | Open reading frame of ToP996 was subcloned with BamHI/PstI into ToP771 |

*: All plasmids coding for fusion proteins of full length ribosomal proteins with epitope tags used in this study complemented the essential functions of the corresponding ribosomal protein genes in S. cerevisiae. In strains solely expressing these epitope tag fusion alleles, in particular Flag-rpS5 and 5xHA-rpS5, no major rRNA processing phenotype or growth phenotype was detected. **: strains solely expressing 5xHA-rpS5-deltaC encoded by vector ToP1156 showed in total RNA pulse labelling-, nucleocytoplasmic fractionation- and ITS1-FISH analyses the same phenotypes as observed in cells expressing solely Flag-rpS5-deltaC .
