# Supplementary material for: A Local Role for the Small Ribosomal Subunit Primary Binder rpS5 in Final 18S rRNA Processing in Yeast
Source: PLoS One. 2010 Apr 19;5(4):e10194. doi: 10.1371/journal.pone.0010194 (PMC2856670; doi:10.1371/journal.pone.0010194)
Supplement: Figure S8 — Yeast strains used in this study. (0.03 MB DOC) [file pone.0010194.s008.doc]

**Table S8: *S. cerevisiae* strains used in this study**

| **ToY** | **name** | **genotype** | **plasmids** | **origin** | **construction** |
| --- | --- | --- | --- | --- | --- |
| 198 | RPS5-shuffle | his3-1,leu2-0,ura3-0,lys2-0, YJR123w::kanMX4 | YCplac33-RPS5 | Ferreira-Cerca et.al., 2005 |  |
| 206 | BY4741 | his3-1,leu2-0,met15-0,ura3-0 |  | Euroscarf |  |
| 207 | BY4742 | his3-1,leu2-0,lys2-0,ura3-0 |  | Euroscarf |  |
| 286 | pGAL-RPS2 | his3-1,leu2-0,ura3-0,met15-0,lys2-0, YGL123w::kanMX4 | YCplac111-pGAL-RPS2 | Ferreira-Cerca et.al., 2005 |  |
| 323 | pGAL-RPS5* | his3-1,leu2-0,ura3-0,lys2-0, YJR123w::kanMX4 | YCplac111-pGAL-RPS5 | Ferreira-Cerca et.al., 2005 |  |
| 1659 | pGAL-RPS5 *(TRP) | his3-1,leu2-0,ura3-0,lys2-0, YJR123w::kanMX4 | ToP1155 (TRP1/Nurseothricin/GAL-RPS5/CEN) | this study | Strain ToY198 was transformed with ToP1155 and positive transformants were selected on Nourseothrycin and galactose containing plates. YCplac33-RPS5 (URA3) negative transformants were selected by growth on 5-FOA. |
| 1739 | pGAL-RPS5 (TRP) RIO2-TAP | his3-1,leu2-0,ura3-0,lys2-0, YJR123w::kanMX4,YNL207W-TAP | ToP1155 (TRP1/Nurseothricin/GAL-RPS5/CEN) | this study | Strain ToY1659 was transformed with PCR product of ToO2316 and ToO2317 from ToP97 (pBS1539) and positive transformants were selected on plates lacking uracil. |
| 1765 | pGAL-RPS5 (TRP) NOB1-TAP | his3-1,leu2-0,ura3-0,lys2-0, YJR123w::kanMX4,YOR056C-TAP | ToP1155 (TRP1/Nurseothricin/GAL-RPS5/CEN) | this study | Strain ToY1659 was transformed with PCR product of ToO2221 and ToO2222 from ToP97 (pBS1539) and positive transformants were selected on plates lacking uracil. |

*: no growth or rRNA processing phenotype could be detected in these strains in permissive conditions (YPG medium) when compared with the By4741 wildtype strain .
